# 360° Diagnostic Tool to Personalize Lifestyle Advice in Primary Care for People With Type 2 Diabetes: Development and Usability Study

**DOI:** 10.2196/37305

**Published:** 2023-03-07

**Authors:** Zeena Harakeh, Iris M de Hoogh, Hilde van Keulen, Gino Kalkman, Eugene van Someren, Pepijn van Empelen, Wilma Otten

**Affiliations:** 1 Department of Child Health TNO, Netherlands Organization for Applied Scientific Research Leiden Netherlands; 2 Department of Microbiology and Systems Biology TNO, Netherlands Organization for Applied Scientific Research Leiden Netherlands; 3 Department of Risk Analysis for Products in Development TNO, Netherlands Organization for Applied Scientific Research Utrecht Netherlands; 4 Department of Sustainable Productivity and Employability TNO, Netherlands Organization for Applied Scientific Research Leiden Netherlands

**Keywords:** type 2 diabetes, diagnostic tool, holistic approach, personalized advice, shared decision-making, health professionals

## Abstract

**Background:**

Various multifaceted factors need to be addressed to improve the health and quality of life of people with type 2 diabetes (T2D). Therefore, we developed a web-based decision support tool that comprises a more holistic diagnosis (including 4 domains: body, thinking and feeling, behavior, and environment) and personalized advice. This 360° diagnostic tool enables people with T2D and health care professionals at the general practice to obtain an overview of the most important T2D-related issues and, subsequently, determine the most suitable intervention for the person with T2D.

**Objective:**

This study aimed to describe the systematic and iterative development and evaluation of the web-based 360° diagnostic tool.

**Methods:**

We defined the requirements for the web-based 360° diagnostic tool based on previously developed tools, a literature review, and inputs from a multidisciplinary team of experts. As part of the conceptualization, we defined 3 requirements: diagnostics; feedback; and advice, consultation, and follow-up. Next, we developed and designed the content for each of these requirements. We evaluated the diagnostic part of the tool (ie, measurement instruments and visualization) with a qualitative design, in a usability study with a think-aloud strategy and interview questions, among 8 people with T2D at a Dutch general practice.

**Results:**

For each of the 4 domains, specific parameters and underlying elements were selected, and measurement instruments (including clinical data and questionnaires) were chosen. Cutoff values were defined to identify high-, middle-, and low-ranking scores, and decision rules were developed and implemented using R scripts and algorithms. A traffic light color visual design was created (profile wheel) to provide an overview of the scores per domain. We mapped the interventions that could be added to the tool and developed a protocol designed as a card deck with motivational interview steps. Furthermore, the usability study showed that people with T2D perceived the tool as easy to use, useful, easy to understand, and insightful.

**Conclusions:**

Preliminary evaluation of the 360° diagnostic tool by experts, health care professionals, and people with T2D showed that the tool was considered relevant, clear, and practical. The iterative process provided insights into the areas of improvement, which were implemented. The strengths, shortcomings, future use, and challenges are also discussed.

## Introduction

### Background

Type 2 diabetes (T2D) is considered a public health problem worldwide. The prevalence rate of diabetes is high and continues to increase [1]. Approximately 537 million adults (aged 20-79 years) live with diabetes, of which the majority (90%) have T2D [2]. T2D contributes to increased mortality and morbidity [1,3]. People with T2D have an increased risk of adverse health- (eg, cardiovascular diseases, neuropathy, and retinopathy), social-, and economic-related consequences [1,4,5]. As multiple factors are related to T2D, it is important to use a holistic approach in the management of T2D to improve the health and quality of life of people with T2D. Health care usually focuses on physical health factors, whereas a holistic approach also includes other factors such as lifestyle, mental health, and socioeconomic environment factors, and as such, demedicalizes societal problems [6,7]. Thus, it is important to develop a tool that enables a person with T2D, together with the health care professional at the general practice, to obtain an overview of the most important T2D-related issues to determine the most suitable management options for the individual. Such a tool may (1) increase and support self-management, empowerment, and informed decision-making for people with T2D and (2) improve communication between health care professionals and people with T2D (including shared decision-making and shared treatment strategy) [6].

Besides physical health factors (eg, familial predisposition, insulin resistance, and obesity), lifestyle-related factors (eg, eating pattern, physical activity, and sedentary behavior), mental health factors (eg, emotional stress, anxiety, and depression), and socioeconomic environment factors (eg, neighborhood deprivation) have also been identified as risk factors of T2D [8-11]. These risk factors not only influence T2D but also affect how individuals experience and manage their T2D [7,12]. Inversely, T2D may affect these factors and, thus, an individual’s quality of life. For example, T2D could induce diabetes-related depression or emotional stress and thus may affect mental health [7,13-16]. Moreover, these factors may also interact with each other. For example, the socioeconomic environment (eg, social networks) may influence physical and mental health [7]. Therefore, it is necessary to consider a holistic approach for T2D that involves all these factors during the diagnosis and treatment to obtain a broad perspective on the health status of individuals with T2D. In addition to the empirical evidence that a holistic approach is well suited for diagnosing and managing T2D, it is also in line with the more recent approach of positive health. This concept covers a more dynamic definition of health, focusing on an individual’s capacity to deal with new situations (resilience and coping) [6].

To develop a tool that encompasses a holistic approach, including comprehensive health status assessments that are subsequently discussed between patient and health care professionals and followed up by interventions, if necessary, we were inspired by the web-based tools of the Power2DM project [17] and the Primus project [18]. The goal of the Power2DM project [17] was to support patients with diabetes by developing a self-management support system and a shared decision-making app for health care professionals and patients. The goal of the Primus project [18] was to improve the health and quality of life of people aged 55-74 years. A web-based screening of behaviors that increase the risk of cardiovascular diseases, depression, or loneliness was developed. Using if-then decision rules, the screening results were used to tailor a written health risk assessment. For instance, individuals were advised to lose weight if their BMI was >25. Next, these results were discussed with a nurse practitioner in primary health care and the person with T2D on how to implement the advice to improve the person’s lifestyle. In the Netherlands, people with T2D are mainly under the care of nurse practitioners supervised by a general practitioner (GP) in primary health care. In addition to the Primus project example, we wanted to develop a tool that visualizes the results of the assessment to facilitate the discussion about it and to support informed and shared decision-making. Some examples are available, such as the “Web Diagram” by Huber et al [6], the Assessment of Burden of Chronic Conditions tool [19], and the “Self-Management Web” by Beck et al [20]. We wanted the visualization to be easily understood, encompassing a holistic approach and motivating discussion of actions. Moreover, to facilitate discussions between patients and health care professionals, it is necessary to offer professional guidance on achieving shared decision-making and to use a directive, client-centered counseling style to elicit behavioral change [21,22]. In summary, it is important that the tool includes the following 3 components: diagnosis (ie, performing the diagnosis: the GP’s assistant performs the measurement and the patient completes the questionnaires); feedback (ie, communicating the diagnosis: the patient receives the feedback and the explanation of the results); and advice, consultation, and follow-up (ie, acting on the diagnosis: discussion during the consultation on improving the results via personalized advice and the patient working on personal goals).

### Objectives

For a more holistic diagnosis and personalized advice approach for people with T2D, we developed a web-based tool, that is, a 360° diagnostic tool. In line with empirical evidence, the positive health concept, and the dynamic definition of health, our holistic approach for T2D includes 4 domains: physical health, mental health, lifestyle behavior, and socioeconomic environment. Moreover, this holistic approach sheds light on the relationships between domains, for example, how lifestyle behavior influences physical health. Diagnosing people with T2D in all 4 domains will facilitate a more personalized approach in which they can successfully face their specific physical, emotional, and social challenges, which includes prioritizing and provisioning tailored interventions to improve their T2D and quality of life. The 360° diagnostic tool is intended as a decision support tool that enables people with T2D and health care professionals in the general practice setting to identify and address relevant factors affecting T2D and its impact on a person to determine the most suitable interventions. In this paper, we describe the systematic and iterative development and evaluation of this web-based 360° diagnostic tool.

## Methods

### Procedure

As part of the web-based 360° diagnostic tool, we defined the requirements for diagnosis; feedback; and advice, consultation, and follow-up.

We based our approach regarding diagnosis, feedback, advice, consultation, and follow-up on the setup of the Primus project [18].

First, to enable a diagnosis, an overview of the possible relevant parameters for each of the 4 domains was made. A team of experts from the Netherlands Organization for Applied Scientific Research (TNO), representing multidisciplinary research fields, and health care professionals acted as an advisory committee in the iterative process of the development of the tool. For each domain, the potential list of parameters and elements was drafted by 3 experts in the field using previously developed tools in the Primus project [18] and Power2DM project [17], a literature review, and expert consultation. Thereafter, 3 other experts from the advisory committee reviewed and complemented the list. Parameters were defined as barriers or risk factors for people with T2D and that are changeable. Subsequently, for each parameter, the underlying elements were identified. Elements were defined as the measurable components of a parameter (for instance, the parameter “blood pressure” consists of the elements “systolic blood pressure” and “diastolic blood pressure”). The final step was to select a measurement instrument to assess each element. A measurement instrument had to fulfill certain criteria, such as (validated and reliable) questionnaires that were easy to complete and as short as possible. Then, the instruments regarding the body domain had to be components of standard clinical care (eg, blood pressure).

Second, to provide feedback on each parameter or underlying element, communicating the diagnosis was an important part of the conceptualization. Therefore, for each measurement instrument (ie, element), we had to decide the cutoff values. These values were based on the literature to identify high-, middle-, and low-ranking scores. Decision rules were developed to combine the elements to rank the main parameters. To implement these decision rules for the elements and parameters in a web-based portal, R scripts and algorithms were developed. We aimed to develop a tool that visualizes the assessment results to facilitate discussion and support informed and shared decision-making between people with T2D and health care professionals in general practice. We wanted the visualization to be easily comprehensible, encompassing all 4 domains and motivating discussion of the actions.

Third, to enable advice, consultation, and follow-up, a protocol was needed to discuss the 360° diagnosis in a structured manner during the consultation with the health care professional. The protocol was developed based on a 3-talk model of shared decision-making [21] and motivational interviewing (MI) [22]. The aim of the 360° diagnosis and profile wheel was to identify and address relevant factors that affect a person with T2D and to obtain personalized advice and the most suitable treatment option. Therefore, we first needed to compile a list of interventions in the Netherlands for all the parameters included in the 360° diagnosis. Subsequently, we needed to use the middle- and low-ranking scores in the profile wheel to decide the parameters for which an intervention could be offered to the individual. Interventions were selected if they could positively alter these parameters. We conducted desk research with inputs from (1) experts of the advisory committee and (2) internet searches. Interventions were included when they could be clearly identified as one distinct intervention and when they were available either nationwide or in 2 regional areas where we intended to evaluate and test the 360° diagnostic tool. Interventions could range from apps (eg, MySugr), websites, local walking initiatives, mindfulness courses, or referral to a dietician. Finally, we compiled a list of intervention options that could be added to the tool to provide personalized advice. Interventions were presented as specific pieces of advice for discussion during the consultation that could improve the middle and low scores of parameters or underlying elements. A follow-up was planned after the first consultation.

To evaluate whether the diagnostic part of the tool (ie, instruments, visualization, and technology) was relevant, acceptable, and usable, we presented the tool to the advisory committee and conducted a qualitative study, that is, a usability study with a think-aloud strategy and evaluative interview questions, among people with T2D at the general practice “Mozaiek” in Rotterdam, the Netherlands (for more information about usability testing, refer to the study by Maramba et al [23]). The prototype of the 360° diagnostic tool presented to both the GP and nurse practitioner at the general practice “Mozaiek” in Rotterdam, the Netherlands, received a positive evaluation and was considered relevant and practical. For the usability study, 8 people with T2D were recruited from the general practice “Mozaiek” in Rotterdam, the Netherlands. Participants were recruited via a nurse practitioner of the general practice, specifically focusing on people with T2D with low socioeconomic status. The inclusion criteria for participation in the study were a diagnosis of T2D and being able to read and speak Dutch. The participants participated individually. The procedure was as follows: the interviewer filled in the biomarker data of the participants in the domain “body.” Participants then completed web-based questionnaires for the other 3 domains (duration of 10-20 minutes). On the basis of these responses, the diagnosis could be calculated and shown in the profile wheel. While filling in the questionnaires and inspecting the graphical feedback in the profile wheel, users were encouraged to think aloud. Subsequently, evaluative interview questions regarding certain topics were asked, for example, whether specific questions or questionnaires posed difficulties as well as regarding the profile wheel, whether they recognized themselves in the feedback, and if the icons were self-explanatory. Thus, the interviewer discussed the visualization (eg, understandable, recognizable, and informative) and the questionnaires (eg, wording, difficulty, and length) with the participants. The total session took 90 minutes. The results of the usability study concerned the relevance, acceptability, and usability of the tool, specifically regarding the evaluation of the measurement instruments and the visualization of the tool.

### Ethical Considerations

The 8 participants in the usability study were informed by the nurse practitioner of the general practice about what was expected of them when participating in the study, and participants verbally provided informed consent. Subsequently, during participation, the interviewer informed each participant again at the beginning and asked if there were any questions. The privacy and anonymity of the participants were guaranteed. As a reward for participating in the study, participants received a voucher of €25 (US $26.08). This usability study was the first phase of a larger project approved by the Medical Ethics Committee Brabant (NL67846.028.18; January 8, 2019).

## Results

### Conceptualization and Development Phase

#### Diagnosis

The first list of parameters and instruments compiled by the first 3 experts included 138 different instruments to assess holistic health, including neurology, metabolic health, motor skills, physical fitness, personality, eating behavior, quality of life, and mental health. This list was further edited by another 3 experts focusing on lifestyle and T2D. The final diagnostic tool included the following 4 domains: body, thinking and feeling, behavior, and the environment. Specific parameters for each of the 4 domains were selected. It was decided that each domain should not have >6 parameters for visualization and comprehensibility reasons (Table 1). The domain “body” included the parameters glucose metabolism, blood pressure, cholesterol, weight, and kidney function. The domain “thinking and feeling” included the parameters perceived health, pain, mental health, perceived stress, and problems with T2D. The domain “behavior” included the parameters alcohol consumption, cigarette smoking, eating patterns, physical activity and exercise, sedentary behavior, and T2D management. The domain “environment” included the parameters family, loneliness, work, income, and housing. Most of the parameters consisted of the underlying elements, as listed in Table 1. For example, regarding the domain “behavior,” the following two underlying elements of the parameter “alcohol consumption” were included: (1) the average number of glasses per day and (2) binge drinking.

To develop a T2D diagnostic and communication tool, we selected measurement instruments to assess each element of the 360° diagnostic tool. It was decided how to measure each of these elements and by whom (eg, which measurement instruments, including their feasibility, accessibility, and burden). The elements included for the domain “body” were measured through clinical data filled in by the health care professional, for example, hemoglobin A_1c_ (HbA_1c_; also referred to as glycated hemoglobin), sober glucose, high-density lipoprotein, and low-density lipoprotein. The parameters or underlying elements of the other 3 domains were measured through questionnaires completed by the person with T2D, for example, the average number of glasses of alcohol per day, perceived stress, and loneliness. Furthermore, the number (of items) and duration of the questionnaires were considered to determine which instruments to use.

**Table 1:** A short overview of the domains, parameters, and underlying elements of the 360° diagnosis.

Domain and parameters		Elements	Instruments
**Body^a^**			
	Glucose metabolism	HbA_1c_^b^, fasting glucose, and 2-hour glucose	Determined from blood samples
	Blood pressure	Diastole and systole	Blood pressure monitor
	Cholesterol	HDL^c^, LDL^d^, total cholesterol, and ratio HDL and triglycerides	Determined from blood samples in a laboratory
	Weight	BMI, waist circumference, and waist-hip ratio	Weighing scale and measuring tape
	Kidney functioning	eGFR^e^ and albuminuria stages	Determined from morning urine in a laboratory
**Thinking and feeling^f^**			
	Perceived health	N/A^g^	A single general health item from the Medical Outcomes Survey Short-Form 36 (SF-36) [24]
	Pain	N/A	One item from the Medical Outcomes Survey Short-Form 36 (SF-36) [24]
	Mental health	N/A	WHO-5^h^ Well-being Index [25]
	Perceived stress	N/A	Perceived Stress Scale [26]
	Problems with diabetes	N/A	PAID-5^i^ [27]
**Behavior^f^**			
	Alcohol consumption	The average number of glasses per day and binge drinking	Five questions regarding frequency and quantity measures of alcohol consumption [28]
	Cigarette smoking	Number of cigarettes and craving	A craving question based on the Fagerström Test for Nicotine Dependence [29] and a question on number of cigarettes [30]
	Eating pattern	Fruit, vegetables, soda, fast-food, and snacks	Questions based on Dutch dietary guidelines [31] and Dietary guidelines for people with Type 2 Diabetes [32]
	Physical activity	N/A	SQUASH (Short Questionnaire to Assess Health-enhancing Physical Activity) [33]
	Sedentary behavior	N/A	Questions based on the Marshall sitting questionnaire [34]
	Diabetes management	Glucose monitoring and medication adherence	Diabetes Self-Management Questionnaire [35]
**Environment^f^**			
	Family	Worries about children and worries about relations	Questions based on the DSM^j^ IV [36] and the Dutch Self-Sufficiency Matrix [37]
	Loneliness	N/A	Questions based on the DSM IV [36] and the Dutch Self-Sufficiency Matrix [37]
	Work	N/A	Questions based on the DSM IV [36] and the Dutch Self-Sufficiency Matrix [37]
	Income	N/A	Questions based on the DSM IV [36] and the Dutch Self-Sufficiency Matrix [37]
	Housing	Neighborhood and house	Questions based on the DSM IV [36] and the Dutch Self-Sufficiency Matrix [37]

^a^The elements of this domain were measured using clinical data filled in by a health care professional.

^b^HbA_1c_: hemoglobin A_1c_.

^c^HDL: high-density lipoprotein.

^d^LDL: low-density lipoprotein.

^e^eGFR: estimated glomerular filtration rate.

^f^The elements of this domain were measured through questionnaires completed by people with type 2 diabetes.

^g^N/A: not applicable.

^h^WHO-5: World Health Organization – five.

^i^PAID-5: Problem Areas in Diabetes Scale – five.

^j^DSM: Diagnostic and Statistical Manual of Mental Disorders.

#### Feedback

##### Cutoff Values and Decision Rules

Decision rules had to be formulated based on cutoff points to identify high-, middle-, and low-ranking scores. We decided to visualize these scores based on a “traffic light model,” which communicated high (green)-, middle (orange)-, and low (red)-ranking scores, where middle- and low-ranking scores identified areas of improvement. For instance, psychological well-being was measured using the World Health Organization (WHO)-5 Well-being Index (WHO-5) [25]. The scores on the WHO-5 range from 0 (absence of well-being) to 100 (maximum well-being), and the cutoff score of ≤50 was used for the screening of depression. Thus, scores of ≤50 were “red,” and scores of >50 were “green.” In this case, no “orange” scores were obtained. Another example is blood pressure, which is shown in Table 2. The cutoff points for the separate elements of diastolic and systolic blood pressure were based on the WHO guidelines [38]. The decision rule for combining the separate elements in the aspect of “blood pressure” was developed by the researchers.

**Table 2:** Overview of the cutoff values and decision rules for blood pressure.

Separate elements					Combined parameter		
Systolic cutoff score	Systolic decision rule	Diastolic cutoff score	Diastolic decision rule	Systolic cutoff score		Diastolic cutoff score	Blood pressure decision rule
<140	Green	<90	Green	<140		<90	Green
140 to <160	Orange	90 to 100	Orange	<140		90 to 100	Orange
≥160	Red	>100	Red	<140		>100	Red
N/A^a^	N/A	N/A	N/A	140 to <160		<90	Orange
N/A	N/A	N/A	N/A	140 to <160		90 to <100	Orange
N/A	N/A	N/A	N/A	140 to <160		≥100	Red
N/A	N/A	N/A	N/A	≥160		<90	Red
N/A	N/A	N/A	N/A	≥160		90 to <100	Red
N/A	N/A	N/A	N/A	≥160		≥100	Red

^a^N/A: not applicable.

##### Visualization of the Tool

**Figure 1:**
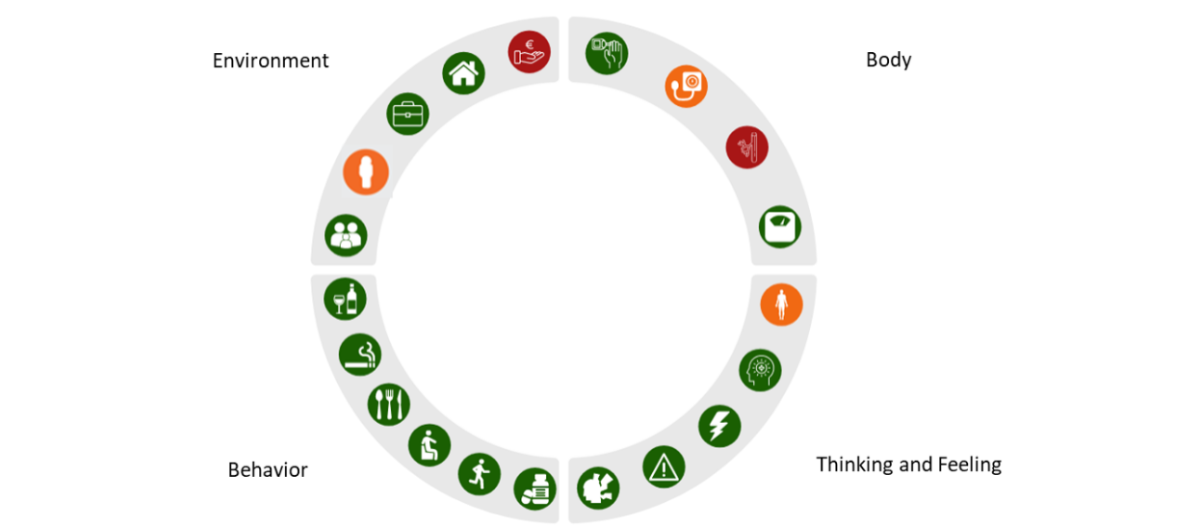
The profile wheel version 1.0 resulting from the 360° diagnosis.

**Figure 2:**
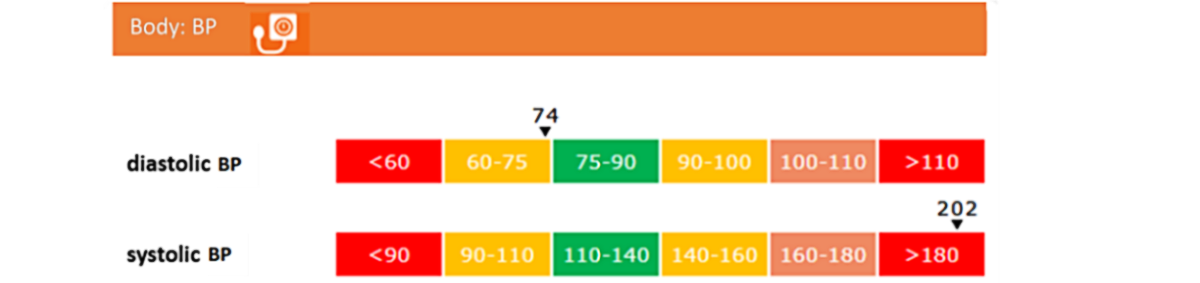
A version 1.0 example of a click-through in the profile wheel for the parameter “blood pressure.” BP: blood pressure.

Next, a visual design was created for the profile wheel (Figure 1) to provide an overview of the scores per domain. We developed this visualization of the 360° diagnostic tool in cooperation with a data scientist expert on visualization. The interaction between the tool and the user involved clicking on the questionnaires, response categories, and icons in the profile wheel. Each domain (ie, body, thinking and feeling, behavior, and environment) was considered equally important. Therefore, the wheel was divided into 4 quadrants of equal sizes. In each quadrant, a maximum of 6 small icons were fitted, reflecting a particular parameter of each domain. For instance, a blood pressure monitor icon represented blood pressure, and the stress icon resembled the outline of a human head with lightning bolts. Depending on the diagnostic data, the icons in the profile wheel were red, orange, or green, reflecting a healthy status or room for improvement. If a parameter consisted of underlying elements, it was possible to click on that particular icon to reveal these elements. When clicking on a particular icon (Figure 2), more specific information was provided. For instance, for blood pressure, scores for systolic and diastolic blood pressure were also shown with traffic light colors indicating cutoff scores. The patient’s score was given as a black colored number, and a small black triangle indicated the corresponding position on the bar.

#### Advice

In the first exploration of the available interventions in the Netherlands, we listed 27 interventions. We classified the interventions into the following categories:

1. Group-based interventions (eg, mindfulness groups to prevent stress)
2. Events (eg, diabetes cafes)
3. Experts and knowledge centers (eg, dietician)
4. Apps (eg, mySugr and stepcounter)
5. Devices (eg, step counter and pulse meter)
6. Programs (eg, combined lifestyle interventions)
7. Knowledge platforms (eg, Dutch Nutrition Center [39]).

We also classified the availability of the interventions into three categories: (1) proximity to the general practice, (2) general practice located within the municipality, and (3) nationwide and web based.

The overview of interventions can be used to discuss options between the patient and health care provider.

#### Consultation and Follow-up

**Figure 3:**
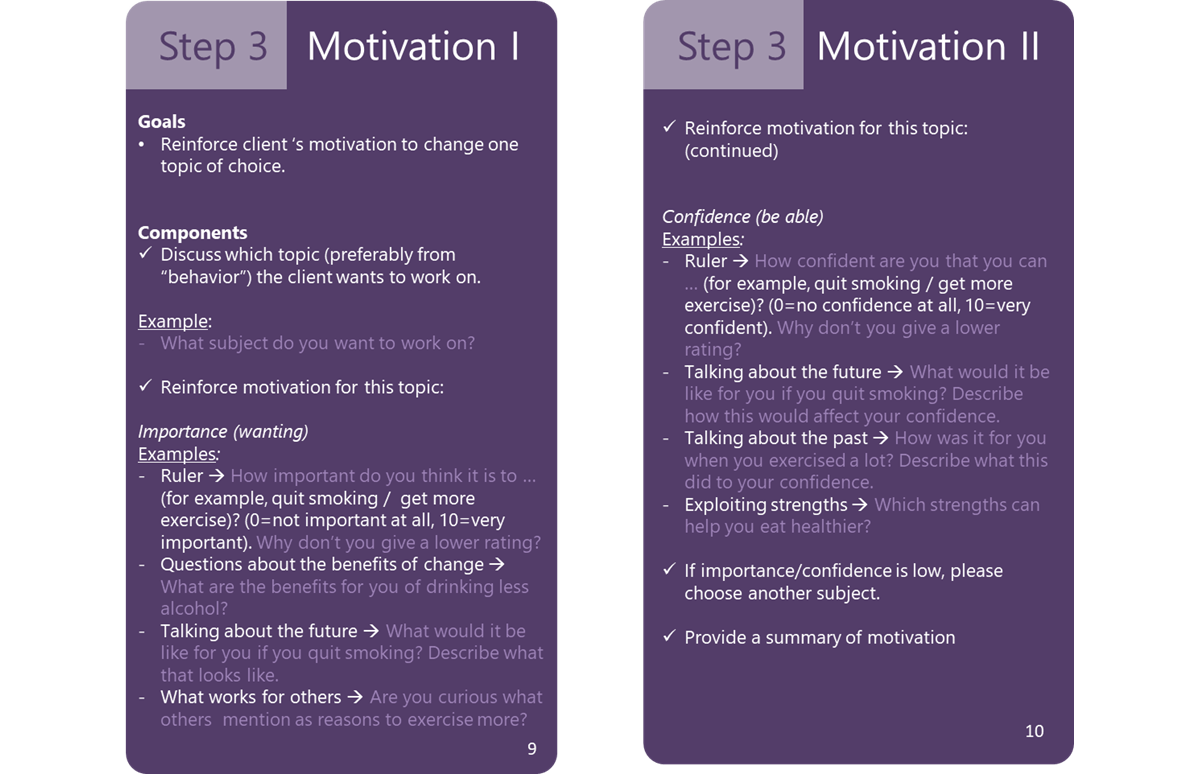
Two example cards of the 360° diagnosis motivational interviewing card deck.

The protocol was drafted by 2 experts on MI based on a Dutch version that represented the MI steps in a set of playing cards [40]. The protocol was designed as a card deck (Figure 3). An expert on shared decision-making reviewed the protocol to check correspondence with shared decision-making principles. A health care professional also reviewed the protocol.

The card deck consisted of the following 15 cards: title page, content, MI basic attitude, MI core skills 1, MI core skills 2, MI core skills 3, diabetes coaching steps overview, step 1 agenda, step 2 current situation I, step 2 current situation II, step 3 motivation I, step 3 motivation II, step 4 planning I, step 4 planning II, and step 5 rounding up. Each skill or step card described the goal, underlying parts, and examples (Figure 3). In step 5 “rounding up,” an intervention was chosen to improve one of the middle (orange)- or low (red)-ranking parameters. Furthermore, a summary was provided about the current situation, the goal (which parameter to improve), the intervention to reach that goal, and possible backup plans and resources to adhere to the intervention.

**Figure 4:**
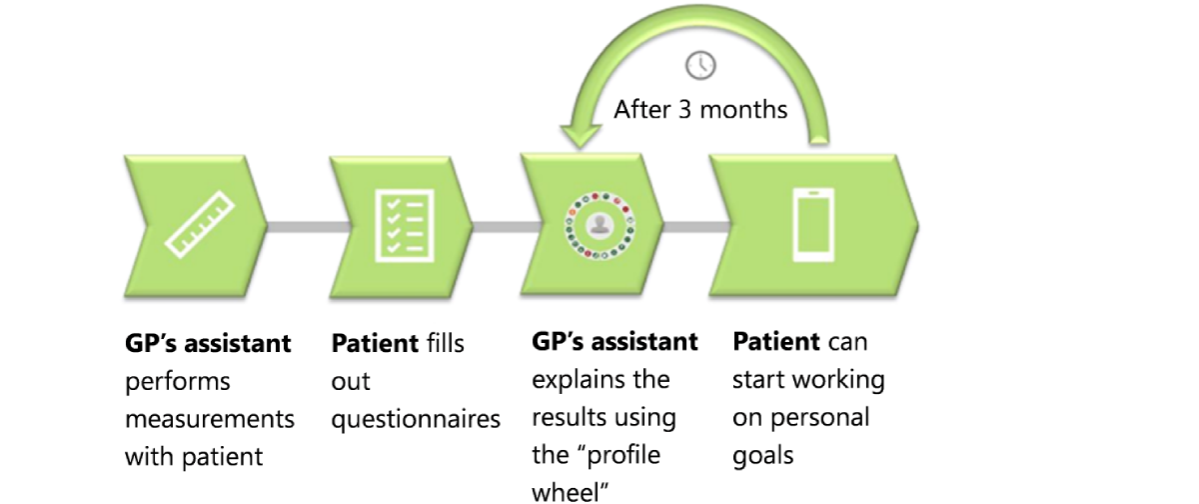
The diagnostic procedure advised to be followed in the general practice for the 360° diagnostic tool. GP: general practitioner.

Follow-up was designed in collaboration with behavioral change experts and health care professionals. Figure 4 shows the 360° diagnostic procedure advised to be followed in general practice. First, the assistant of the GP (eg, nurse practitioner) measures the patient’s clinical data in the body domain. Second, the patient completes the questionnaires for the other 3 domains. In the consultation, the diagnosis represented in the profile wheel is discussed using the protocol, and an intervention is chosen. After approximately 3 months, a second 360° diagnosis is performed by the GP’s assistant to check how the patient is doing and to discuss whether possible improvements have been made. The second 360° diagnosis is an important form of feedback for both the health care professional and the patient.

### Evaluation Phase

#### Overview of the Participants

In total, 5 males and 3 females participated in the usability study, and their ages ranged from 34 to 70 years (mean 54.25, SD 10.35 years). Five participants had lower education, 1 had middle education, 1 had higher education, and 1 participant’s educational background was unknown. Half of the participants were Dutch. The duration of T2D ranged from 2 weeks to 4 years, and for 1 participant, the duration was unknown. Five participants used medication for T2D, and for 2 participants, it was unknown.

#### Evaluation of the Measurement Instruments of the Tool

Half of the participants took approximately 12 minutes to answer all the questions included in the 360° diagnostic tool. One of the reasons that some participants took relatively more time to answer the questionnaires was that the system was too slow owing to bad internet connectivity. In general, the participants understood the questions in the questionnaire and were able to answer them easily. They did not perceive the questions to be difficult, strange, or unexpected. Of all the included questionnaires, the stress questionnaire took the most time to complete because stress was perceived as a more complex topic by participants and because it consisted of more questions.

The first questionnaires in the 360° diagnostic tool were more generic, but the participants were originally expecting questions about diabetes. In addition, it is not always clear for participants whether questions (eg, regarding pain) were asked in general or in relation to T2D. In addition, explanations were missing regarding how certain parameters, such as loneliness, were related to diabetes. Therefore, we revised the structure of the questionnaires. We also added a brief introduction to the questionnaires so that participants would understand why we asked these questions about a certain parameter that was not obviously related to diabetes.

In terms of response categories, participants did not perceive the organization of the response categories of some of the questions assessing the parameter “problems with diabetes” as logical. Therefore, they were adjusted such that these response categories ranged from “not a problem” to “a serious problem.” Moreover, some questions required additional response categories. For example, “no relationship,” “having a relationship, but not living together,” and “living together” were missing as response categories to the question about marital status. In addition, some questions needed to be adjusted to be applicable to people with T2D. For example, questions about diet were adjusted to fit the recommended dietary pattern. Furthermore, routing was lacking for some questions. For example, participants who indicated they did not drink alcohol still received a follow-up question on how many glasses of alcohol they consumed during the week or weekend, and they needed to fill in 0 glasses to be able to continue with the questionnaire.

The number of questions was deemed to be adequate. Although some participants indicated that for some domains and parameters, more questions could have provided a more detailed view of their context or situation, for instance, parameters measured with only 1 question, such as loneliness or income. However, we decided to keep the questionnaires short and did not include additional questions. In addition, more information and nuances can be discussed in further detail during the consultation with the health care professional.

In conclusion, positive feedback on the questionnaires affirmed our choice and the length of the questionnaires for the 360° diagnostic tool. The evaluations suggested improvements to the structure and introduction of the questionnaires and response categories for some questions (including routing). These points for improvement were easily adjusted and integrated into the 360° diagnostic tool.

#### Evaluation of the Visualization and Feedback of the Tool

**Figure 5:**
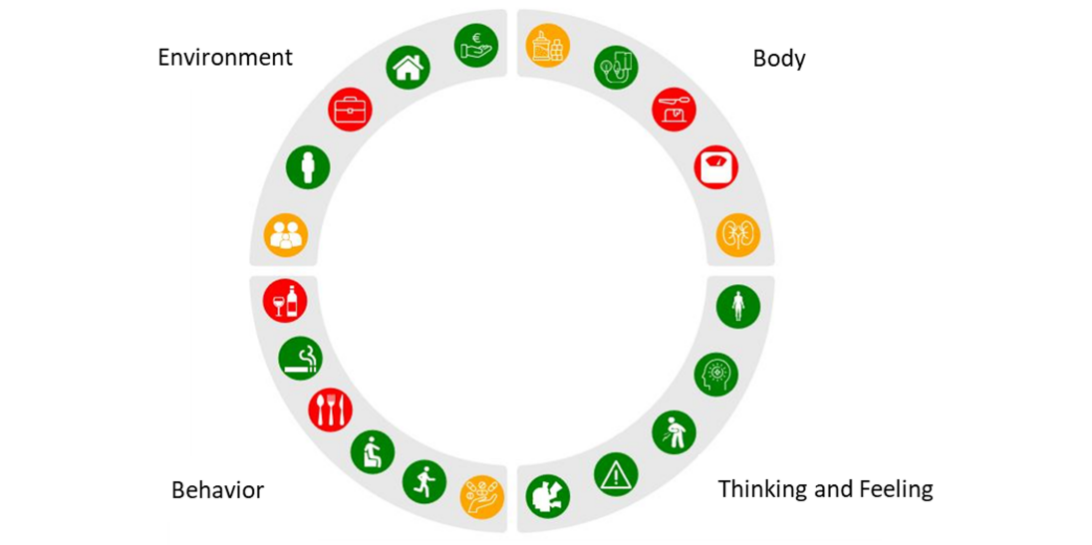
The adjusted profile wheel after evaluation of the usability study.

In general, the participants perceived the profile wheel visualization, including the icons presented, as pleasant. The profile wheel did not contain much information and was perceived as useful. Participants indicated that it helped them keep track of their health and provided personalized results that could be discussed and analyzed with their health care provider. However, complex language and the use of technical terminology caused some problems in understanding the profile wheel. As a result, we changed the text accordingly. For example, “glucose” was replaced by “blood sugar.” The visualizations and colors in the profile wheel were perceived as clear and corresponded with the participant’s health status. Participants understood the traffic light colors (green, orange, and red) directly and had an overall impression of each domain. The participants reported some icons that were perceived as unclear and confusing. For instance, they did not recognize that the blood pressure icon represented a blood pressure monitor or that the glucose icon represented a finger prick. As a result, we redesigned these icons into a clearer icon of a blood pressure monitor and a glucose icon representing sugar (Figure 5).

Clicking through icons was considered nice and informative. However, the participants expected that every icon would have a click-through, and this was not the case. Adjustments were made to the profile wheel so that it was possible to click on each icon. Furthermore, when clicking on the icon, it was not always clear what the depicted values meant and how to interpret the results. However, this needed to be optimized. Furthermore, for 1 participant, the profile wheel could not be shown because the questionnaires were not fully answered; however, it was resolved in a newer version. The profile wheel in the newer version shows gray icons if certain questions remain unanswered. The usability study also showed that programming (underlying scripts and algorithms) required further work. For example, the icon for cholesterol was red, whereas the underlying data did not imply red classification. In addition, the technical system (ie, the portal of the 360° diagnostic tool) did not always work as intended and was sometimes slow on the time scale of minutes.

In conclusion, the visualization of the profile wheel (including click-through icons) was evaluated positively regarding relevance, ease of use, and usability for the 360° diagnostic tool. The evaluations showed some suggestions for improvements regarding simpler language (including avoiding technical terminologies) in the feedback, clearer and click-through icons, and debugging the underlying scripts and algorithms in the profile wheel. These points of improvement were incorporated into the updated version of the profile wheel.

## Discussion

### Principal Findings

The treatment of T2D could benefit from a more holistic diagnosis, which provides insights into the main underlying T2D-related issues for an individual, shared decision-making, and puts the patient first. Therefore, a 360° diagnostic tool has been developed for this purpose. A preliminary evaluation of our tool by a small number of experts and health care professionals showed that the tool was relevant, clear, and practical. These experts and health care professionals only commented on the tool and not on its implementation process. People with T2D perceived the tool, including the measurement instruments and visualization, as easy to use, useful, easy to understand, and insightful.

In developing the 360° diagnostic tool, a systematic and iterative approach was followed, which has several advantages. First, a broad range of stakeholders was involved, including experts from multiple domains and health care providers. This broad consultation was combined with extensive desk research. This contributed to a comprehensive overview of the factors related to T2D and enabled their prioritization. Second, by using a systematic approach, the development process of the tool is transparent, and all content and design choices are well documented. Third, by adopting an iterative approach, areas of improvement could be identified early in the process. This iterative approach involved multiple consultations with experts in the field as well as presenting the tool to health care providers. The patients were included in the process during the usability study.

The main points of improvement in the usability study included the logic of some of the answer options, amending explanation texts for both the questionnaires and the profile wheel, and adjusting some of the icons used in the profile wheel. In addition, certain technical parameters needed to be resolved. The iterative nature of the development process helped identify gaps and issues and provided an opportunity to resolve them before the tool was implemented in primary practice. For instance, as some icons were not clear to the patients, a newer version of the profile wheel was developed (Figure 5). Finally, involving health care providers and patients early in the process facilitated the future implementation of the tool.

In the development process of the 360° diagnostic tool, choices were made regarding the selection of relevant domains and their underlying parameters and elements. For example, the Web Diagram by Huber et al [6], which can be considered a similar approach, also includes the “spiritual domain.” We decided not to include this domain, as we feel it is partly covered by the “mental health” domain, and is more fitting as part of MI in finding potential solutions during the consultation, instead of as a diagnostic criterion per se. In the selection of domains, the parameter and element focus was on the known changeable factors related to T2D. As such, the 360° diagnostic tool not only visualizes the burden of disease but also provides the opportunity for tailored and actionable recommendations via shared decision-making. In the usability test, no specific domains, parameters, or elements were deemed missing by people with T2D or health care professionals. In addition, the domains, parameters, and elements included in our tool largely overlap with the standard set of outcomes that reflect what matters most to patients as proposed by the International Consortium for Health Outcomes Measurement [41], except for specific diabetes-related complications and medication for comorbidities. Diabetes-related complications, however, were generically included in our tool by assessing compounding problems.

Our tool is also unique in combining subjective (questionnaires) with objective (biomarkers, body composition, etc) measurements, whereas other tools such as the Self-Management Web by Beck et al [20], the Assessment of Burden of Chronic Conditions tool [19], and the Web Diagram by Huber et al [6] only include subjective measurements and are therefore more vulnerable to bias. In addition, the questionnaires included in the 360° diagnostic tool consisted mostly of well-established and validated questionnaires, which allowed for the benchmarking of individual scores to subgroup or population averages. What all tools have in common is the holistic view of health, their supporting function in the conversation between health care providers and patients, and their focus on shared decision-making. The importance of a comprehensive approach in treating chronic diseases such as T2D has also been recognized by the WHO [42], which stipulates that several factors influence treatment adherence and that these factors may interact with each other and, as such, affect both adherence and metabolic control.

Another strength of our tool is that it is visual, which contributes to a better understanding, is more persuasive than text, and helps to reveal underlying patterns [43]. It is also more appropriate for people with low socioeconomic status (which is related to health literacy). The usability study included people with low socioeconomic status who reported that the tool was clear and easy to use. Fit for use by people with low socioeconomic status or health literacy is very relevant because chronic conditions such as T2D are more prevalent among them, and therefore, treatment adherence is a greater challenge [42].

### Limitations

First, a shortcoming of this study is that only 1 primary care practice was involved. The development process and usability study could have been improved by including health care professionals and patients from multiple health care practices to generate a more representative sample and increase the generalizability of the results. Second, the usability study only involved an evaluation of the questionnaires and the visualization via an interview. The protocol for health care providers to discuss the tool and the intervention toolbox needs to be evaluated in future research. In addition, further research is required to assess the feasibility and cost-effectiveness of implementing the tool in a real-world primary care setting.

### Implications for Future Use

The 360° tool and intervention toolbox should be continuously updated with changing guidelines. We also aim to further develop the tool to increase its usability and value in primary care. First, the profile wheel currently shows values for a single assessment but monitoring changes over time for individual patients needs to be incorporated as a part of the 360° diagnostic procedure in practice (Figure 4). Monitoring the use of the profile wheel can be valuable for tracking progress and assessing treatment effectiveness in a more holistic way. Therefore, we aim to visualize its changes over time for individual patients.

Second, the intervention overview has not yet been integrated into the digital 360° tool. We aim to include an intervention toolbox and algorithms linking the diagnosis to appropriate interventions within the tool used in this study to facilitate and support people with T2D to undertake action based on the diagnosis. However, offline interventions are often location specific, which demands situation-specific identification of fitting interventions. Future development of the intervention toolbox will include situation-specific decisions for the appropriate interventions to be added to the toolbox along with the domains and parameters they are expected to be effective for. These decision algorithms should be continuously updated based on the data collected after their implementation in primary care.

Finally, the 360° tool has been developed specifically for people with T2D, but as also shown by Boudewijns et al [19], there is a large overlap in the factors influencing chronic diseases. Thus, after adaptation, our tool may be translated to other chronic diseases or potentially for preventive purposes. Therefore, we aim to adjust the backbone of the 360° tool in such a way that it becomes modular, such that new domains, parameters, or elements can be added or removed more easily to adapt the tool for other chronic diseases, such as cardiovascular risk management, or even completely new diagnostic purposes, such as implementation in pregnancy monitoring. If the tool is used for other purposes, it is still necessary to consult the relevant experts and perform a literature review to identify the missing parameters or elements.

### Conclusions

The web-based 360° diagnostic tool is a decision support tool to identify the main underlying T2D-related issues for an individual, to determine the most suitable interventions by including a holistic diagnosis, and to facilitate shared decision-making between a person with T2D and the health care professionals in primary care. Preliminary evaluation of the 360° diagnostic tool by a small number of experts, health care professionals, and people with T2D showed that the tool is considered relevant, clear, and practical. The iterative development process provided insights into the areas of improvement, which were implemented later. In addition, the usability and value of the tool will benefit from further research (eg, feasibility, impact, and cost-effectiveness) and development (eg, monitoring and visualizing changes over time, integrating interventions as part of the tool, and adapting it for other chronic diseases and diagnostic or preventive purposes). Therefore, this tool may lead to a more personalized treatment strategy that may result in better health outcomes and quality of life.

## Abbreviations

GP: general practitioner
HbA_1c_: hemoglobin A_1c_
MI: motivational interviewing
T2D: type 2 diabetes
TNO: Netherlands Organisation for Applied Scientific Research
WHO: World Health Organization

## References

[1] (2022). Diabetes. World Health Organization.

[2] Diabetes Atlas. International Diabetes federation (IDF).

[3] Overbeek JA, van der Heijden AW, Herings RM, Nijpels G (2017). [Prevalence of diabetes mellitus in the Netherlands more than doubled in the period 1999-2014]. Ned Tijdschr Geneeskd.

[4] American Diabetes Association (2013). Economic costs of diabetes in the U.S. in 2012. Diabetes Care.

[5] van Ommen B, Wopereis S, van Empelen P, van Keulen HM, Otten W, Kasteleyn M, Molema JJ, de Hoogh IM, Chavannes NH, Numans ME, Evers AW, Pijl H (2017). From diabetes care to diabetes cure-the integration of systems biology, eHealth, and behavioral change. Front Endocrinol (Lausanne).

[6] Huber M, van Vliet M, Giezenberg M, Winkens B, Heerkens Y, Dagnelie PC, Knottnerus JA (2016). Towards a 'patient-centred' operationalisation of the new dynamic concept of health: a mixed methods study. BMJ Open.

[7] Mendenhall E, Kohrt BA, Norris SA, Ndetei D, Prabhakaran D (2017). Non-communicable disease syndemics: poverty, depression, and diabetes among low-income populations. Lancet.

[8] Lindekilde N, Rutters F, Erik Henriksen J, Lasgaard M, Schram MT, Rubin KH, Kivimäki M, Nefs G, Pouwer F (2021). Psychiatric disorders as risk factors for type 2 diabetes: an umbrella review of systematic reviews with and without meta-analyses. Diabetes Res Clin Pract.

[9] Mezuk B, Chaikiat Å, Li X, Sundquist J, Kendler KS, Sundquist K (2013). Depression, neighborhood deprivation and risk of type 2 diabetes. Health Place.

[10] Symptoms and causes of diabetes. National Institute of Diabetes and Digestive and Kidney Diseases (NIDDK).

[11] Pouwer F, Kupper N, Adriaanse MC (2010). Does emotional stress cause type 2 diabetes mellitus? A review from the European Depression in Diabetes (EDID) Research Consortium. Discov Med.

[12] Kalra S, Jena BN, Yeravdekar R (2018). Emotional and psychological needs of people with diabetes. Indian J Endocrinol Metab.

[13] Ali S, Stone MA, Peters JL, Davies MJ, Khunti K (2006). The prevalence of co-morbid depression in adults with type 2 diabetes: a systematic review and meta-analysis. Diabet Med.

[14] Hendrieckx C, Halliday JA, Beeney LJ, Speight J (2022). Diabetes and emotional health: a practical guide for healthcare professionals supporting adults with type 1 and type 2 diabetes. 2nd edition. Diabetes UK.

[15] Hoogendoorn C, Shapira A, Roy J, Kane N, Gonzalez J, Delamater AM, Marrero DG (2020). Diabetes distress and quality of life in adults with diabetes. Behavioral Diabetes: Social Ecological Perspectives for Pediatric and Adult Populations.

[16] (2013). NDF Richtlijn Signalering en monitoring van depressieve klachten bij mensen met diabetes. Nederlandse Diabetes Federatie.

[17] Glachs D, Namli T, Strohmeier F, Rodríguez Suárez G, Sluis M, Delgado-Lista J, Sont JK, de Graaf AA, Salzsieder E, Vogt L (2021). A predictive model-based decision support system for diabetes patient empowerment. Stud Health Technol Inform.

[18] van Dijk DJ, Crone MR, van Empelen P, Assendelft WJ, Middelkoop BJ (2017). Favourable outcomes of a preventive screening and counselling programme for older people in underprivileged areas in the Netherlands: the PRIMUS project. Prev Med Rep.

[19] Boudewijns EA, Claessens D, van Schayck OC, Keijsers LC, Salomé PL, in 't Veen JC, Bilo HJ, Gidding-Slok AH (2020). ABC-tool reinvented: development of a disease-specific 'Assessment of Burden of Chronic Conditions (ABCC)-tool' for multiple chronic conditions. BMC Fam Pract.

[20] Beck D, Been-Dahmen J, Peeters M, Grijpma JW, van der Stege H, Tielen M, van Buren M, Weimar W, Ista E, Massey E, van Staa A (2019). A nurse-led self-management support intervention (ZENN) for kidney transplant recipients using intervention mapping: protocol for a mixed-methods feasibility study. JMIR Res Protoc.

[21] Elwyn G, Durand MA, Song J, Aarts J, Barr PJ, Berger Z, Cochran N, Frosch D, Galasiński D, Gulbrandsen P, Han PK, Härter M, Kinnersley P, Lloyd A, Mishra M, Perestelo-Perez L, Scholl I, Tomori K, Trevena L, Witteman HO, Van der Weijden T (2017). A three-talk model for shared decision making: multistage consultation process. BMJ.

[22] Miller WR, Rollnick S (2012). Motivational Interviewing: Helping People Change. 3rd edition.

[23] Maramba I, Chatterjee A, Newman C (2019). Methods of usability testing in the development of eHealth applications: a scoping review. Int J Med Inform.

[24] Ware JE, Sherbourne CD (1992). The MOS 36-item short-form health survey (SF-36). I. Conceptual framework and item selection. Med Care.

[25] Topp CW, Østergaard SD, Søndergaard S, Bech P (2015). The WHO-5 Well-Being Index: a systematic review of the literature. Psychother Psychosom.

[26] Cohen S, Kamarck T, Mermelstein R (1983). A global measure of perceived stress. J Health Soc Behav.

[27] McGuire BE, Morrison TG, Hermanns N, Skovlund S, Eldrup E, Gagliardino J, Kokoszka A, Matthews D, Pibernik-Okanović M, Rodríguez-Saldaña J, de Wit M, Snoek FJ (2010). Short-form measures of diabetes-related emotional distress: the Problem Areas in Diabetes Scale (PAID)-5 and PAID-1. Diabetologia.

[28] Lemmens P, Tan ES, Knibbe RA (1992). Measuring quantity and frequency of drinking in a general population survey: a comparison of five indices. J Stud Alcohol.

[29] Fagerström KO (1991). Towards better diagnoses and more individual treatment of tobacco dependence. Br J Addict.

[30] Mudde AN, Willemsen MC, Kremers SP, de Vries H (2000). Meetinstrumenten voor onderzoek naar roken en stoppen met roken.

[31] Wat staat er in de Schijf van Vijf, en wat niet?. Voedingscentrum.

[32] Eten met diabetes. Diabetes Fonds.

[33] Wendel-Vos GC, Schuit AJ, Saris WH, Kromhout D (2003). Reproducibility and relative validity of the short questionnaire to assess health-enhancing physical activity. J Clin Epidemiol.

[34] Marshall AL, Miller YD, Burton NW, Brown WJ (2010). Measuring total and domain-specific sitting: a study of reliability and validity. Med Sci Sports Exerc.

[35] Schmitt A, Kulzer B, Ehrmann D, Haak T, Hermanns N (2022). A self-report measure of diabetes self-management for type 1 and type 2 diabetes: the diabetes self-management questionnaire-revised (DSMQ-R) – clinimetric evidence from five studies. Front Clin Diabetes Healthc.

[36] Bell CC (1994). DSM-IV: diagnostic and statistical manual of mental disorders. JAMA.

[37] Fassaert T, Lauriks S, van de Weerd S, Theunissen J, Kikkert M, Dekker J, Buster M, de Wit M (2014). Psychometric properties of the Dutch version of the self-sufficiency matrix (SSM-D). Community Ment Health J.

[38] Whitworth JA, World Health Organization‚ International Society of Hypertension Writing Group (2003). 2003 World Health Organization (WHO)/International Society of Hypertension (ISH) statement on management of hypertension. J Hypertens.

[39] Voedingscentrum.

[40] van Merendonk S (2014). Waaier Motiverende Gespreksvoering.

[41] ICHOM Diabetes in Adults Working Group (2019). Type 1 and Type 2 Diabetes in Adults: data collection reference guide. Version 1.0.0. International Consortium for Health Outcomes Measurement.

[42] (2003). Adherence to long-term therapies: evidence for action. World Health Organization.

[43] Dur BI (2014). Data visualization and infographics in visual communication design education at the age of information. J Arts Humanit.

